# Association between short-term heart rate variability and blood coagulation in patients with breast cancer

**DOI:** 10.1038/s41598-021-94931-w

**Published:** 2021-07-29

**Authors:** Lingling Wang, Jingfeng Wang, Peng Li, Xiangzhi Wang, Shuang Wu, Bo Shi

**Affiliations:** 1 School of Medical Imaging, Bengbu Medical College, Bengbu, 233030 Anhui China; 2 Anhui Key Laboratory of Computational Medicine and Intelligent Health, Bengbu Medical College, Bengbu, 233030 Anhui China; 3 Division of Sleep and Circadian Disorders, Brigham and Women’s Hospital, Boston, MA 02115 USA; 4 Division of Sleep Medicine, Harvard Medical School, Boston, MA 02115 USA; 5 Department of Radiation Oncology, First Affiliated Hospital, Bengbu Medical College, Bengbu, 233004 Anhui China

**Keywords:** Biophysics, Cancer, Computational biology and bioinformatics, Biomarkers

## Abstract

The purpose of this study was to investigate the relationship between heart rate variability (HRV), a non-invasive tool for evaluating autonomic function, and routine coagulation indices (RCIs) in patients with breast cancer (BC). Forty-six BC patients were enrolled in this study. Blood biochemistry tests were performed to extract RCIs, including prothrombin time (PT), activated partial thromboplastin time (APTT), and thrombin time (TT). Five-minute electrocardiograms were collected for analysis of HRV parameters (SDNN, RMSSD, LF, HF, LF n.u., HF n.u., LF/HF). Multiple linear regression models examined the relationship of HRV parameters with RCIs. RMSSD, LF n.u., HF n.u., LF/HF were significantly associated with PT. Specifically, the value of PT increased by 0.192 ± 0.091 or 0.231 ± 0.088 s, respectively for each 1 standard deviation (SD) increase in RMSSD or HF n.u.; it increased by 0.230 ± 0.088 or 0.215 ± 0.088 s, respectively for each 1 − SD decrease in LF n.u. or ln (LF/HF) (all *P* < 0.05). RMSSD was significantly associated with APTT, i.e., the value of APTT increased by 1.032 ± 0.470 s for each 1 − SD increase in RMSSD (*P* < 0.05). HRV parameters were associated with RCIs in patients with BC. These observations suggest that the autonomic nervous system and coagulation indices in BC patients are linked, potentially explaining the reason that they are both associated with the prognosis.

## Introduction

Breast cancer (BC) patients often have disrupted coagulation function^1–3^. Prior studies found that, compared with patients with other benign breast diseases, BC patients demonstrated changes in routine coagulation indices (RCIs), such as decreased prothrombin time (PT), decreased activated partial thromboplastin time (APTT), and decreased thrombin time (TT)^4^. Besides, regular coagulation tests and the use of appropriate anticoagulant drugs in BC patients were reported to help reduce the occurrence of thrombotic disease and tumour metastasis^5,6^.

Autonomic nerve activity also plays a significant role in tumorigenesis of BC patients^7,8^. As a quantitative assessment of the function of cardiac autonomic nervous system (ANS), heart rate variability (HRV) was found to be associated with survival and tumour stage in BC patients. For example, Arab et al. found that greater HRV reduction was associated with more profound tumour stage^9^. Giese-Davis et al. found that higher high-frequency (HF) power of HRV significantly predicted longer overall survival^10^.

Other studies also observed that certain associations between ANS function and coagulation existed in cancer patients. Activation of the sympathetic nervous system (SNS) has been reported to initiate coagulation by increasing blood fibrinogen (FIB), clotting factor VIII, von Willebrand factor, and platelet activity^11,12^. Mais et al. showed that SNS activation through adrenergic agonists that stimulates adrenergic responses could induce the procoagulant state in healthy individuals and lead to the shortening of APTT and the increase of D-dimer and prothrombin fragment F1 + 2^13^. Westerloo et al. showed that vagus nerve stimulation can inhibit the activation of coagulation and fibrinolysis system induced by dose of lipopolysaccharide in rats through cholinergic anti-inflammatory pathway^14^. Based on HRV analysis, Wang et al. reported associations between frequency-domain HRV parameters and FIB level in gastric cancer (GC) patients^15^. Shi et al. studied nonlinear HRV characteristics of GC patients and found that increased short-range temporal correlations, decreased asymmetry, and increased irregularity of heartbeat fluctuations were associated with elevated FIB level^16^. However, it is yet to verify whether these changes in HRV are also linked to coagulation function in BC patients. The aim of this work is to investigate the association between short-term HRV parameters and RCIs in BC patients. To this end, we enrolled 46 patients who were first diagnosed with BC. Prior to surgical treatments, 5-min resting electrocardiogram (ECG) was recorded to enable HRV analysis, and fasting venous blood was collected for performing coagulation assay. Bivariate correlation of RCIs (including PT, APTT, and TT) with time- and frequency-domain HRV parameters was performed followed by linear regression models to account for potential confounding factors including age and body mass index (BMI).

## Methods

### Subjects

BC patients admitted to The First Affiliated Hospital of Bengbu Medical College between November 2018 and June 2019 were enrolled. All patients were female and were first diagnosed with BC by needle biopsy and/or pathological examination, and none of them had undergone any treatments (such as radiotherapy, chemotherapy, or surgery). Patients with primary diseases of vital organs (such as heart, lung, liver and kidneys), severe infections, long-term use of hormones, a history of anticoagulant thrombolytic therapy in the past three months, or was breastfeeding or pregnancy were excluded. This study was approved by the Clinical Medical Research Ethics Committee of The First Affiliated Hospital of Bengbu Medical College (Bengbu, Anhui, China) (registration number: 2019KY031). The experiments were conducted in strict accordance with the ethical standards set forth in the 1964 Declaration of Helsinki and its later amendments. Ultimately, 46 BC patients were included in this study. All the patients were informed of the objectives, contents, latent risks, and signed written informed consents.

### Data collection

Single-lead ECG data were collected from each patient for 5 min using a Micro-ECG recorder (Healink-R211B, HeaLink Ltd., Bengbu, China). The Healink ECG recorder has three sampling modes: 100 Hz, 200 Hz, and 400 Hz. Ziemssen et al. recommended that the sampling rate for HRV analysis be no lower than 250 Hz^17^; therefore, we chose a sampling rate of 400 Hz. During the test, subjects were in a sitting position. Two disposable Ag/AgCl ECG electrodes (Junkang Ltd., Shanghai, China) were adhered at the V6 positions for use.

Fasting venous blood was collected from all patients in the morning before breast-conserving surgery and modified radical mastectomy. The time interval between ECG measurement and blood test was within 2 days. The blood samples were combined with sodium citrate to prevent coagulation; the ratio of anticoagulant volume to blood volume was 1:9. PT, APTT, and TT were analysed using an automatic coagulation analyser (Sysmex CS51000, Sysmex Corporation, Kobe, Japan). PT, APTT and TT were all detected by the solidification method. All the test reagents used above were the original reagents imported with the instrument.

### Heart rate variability analysis

The collected ECG data were transmitted to a computer via Bluetooth. The R-to-R interval (RRI) time series were extracted by ECGViewer software (version 2.0, https://www.healink.ltd, HeaLink Ltd., Bengbu, China) with visual inspections afterwards to remove artefacts (such as ectopic beats). Seven of them had fewer than three ectopic beats, and one had a rate of less than 6%. The number of ectopic beats in each subject was less than 10% of all beats. Then, HRV time- and frequency-domain analyses were performed.

The time-domain parameters included the standard deviation of all normal-to-normal (NN) intervals (SDNN) and the root mean square of the normal difference between successive NN intervals (RMSSD)^18^.

The frequency-domain parameters included the low-frequency (LF; 0.04–0.15 Hz) power, the high-frequency (HF; 0.15–0.40 Hz) power, the LF power in normalized units (LF n.u.), the HF power in normalized units (HF n.u.), and the ratio of LF power to HF power (LF/HF). Prior to frequency-domain analysis, the RRI time series was resampled at 4 Hz using cubic spline interpolation^19^. The fast Fourier transform (FFT) with the Welch’s periodogram method (with 150 s window width and 50% overlapping window) was applied to estimate the power spectral density of the RRI time series^20^. The spectrum is obtained by averaging the spectra of these overlapping segments for decreasing the variance of the FFT spectrum.

The analyses of the above parameters were performed with Kubios HRV Premium software (version 3.1.0, https://www.kubios.com, Kubios Oy, Kuopio, Finland)^21^.

### Statistical analysis

Bivariate Pearson correlation was used to analyse the relationship of HRV parameters and RCIs. Significant correlation was considered if *P* < 0.05. Multiple linear regression models were performed to account for potential confounding effects, including age and BMI. The Benjamini–Hochberg procedure was used to control for the false discovery rate (FDR) across multiple comparisons. Statistical significance was accepted if the FDR-corrected *P* < 0.05. SPSS statistical software (ver. 22.0, IBM Corp., Chicago, Illinois, United States of America) was used for the above analysis.

## Results

**Figure 1:**
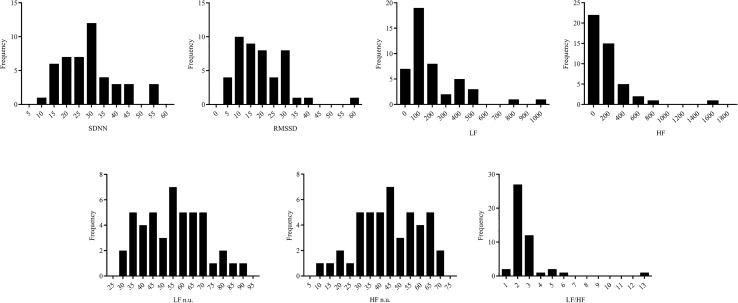
Histograms of HRV parameters (SDNN, RMSSD, LF, HF, LF n.u., HF n.u., and LF/HF).

The histograms of HRV parameters (SDNN, RMSSD, LF, HF, LF n.u., HF n.u., and LF/HF) are shown in Fig. 1. Histograms of LF, HF, and LF/HF showed obvious right skewness, they were expressed as median [interquartile range] and a natural log transform was applied prior to further analysis. Table 1 summarizes the demographics, RCIs, and HRV parameters of all participants.

**Table 1:** Basic data of the studied BC patients.

Variables	Values
*N* (female)	46 (46)
Age (years)	52.6 (11.0)
BMI (kg/m^2^)	24.4 (3.3)
PT (s)	11.1 (0.6)
TT (s)	19.4 (2.2)
APTT (s)	25.1 (3.2)
SDNN (ms)	28.8 (11.7)
RMSSD (ms)	18.8 (10.8)
LF (ms^2^)	117 [202]
HF (ms^2^)	108 [195]
LF n.u. (%)	55.6 (15.1)
HF n.u. (%)	44.4 (15.0)
LF/HF	1.301 [1.313]

Values are expressed as the mean (SD) or median [interquartile range].

*BMI* body mass index, *PT* prothrombin time, *TT* thrombin time, *APTT* activated partial thromboplastin time, *SDNN* standard deviation of normal-to-normal intervals, *RMSSD* the root mean square of successive heartbeat interval differences, *LF* low-frequency power, *HF* high-frequency power, *LF n.u.* low-frequency power in normalized units, *HF n.u.* high-frequency power in normalized units, *LF/HF* the ratio of low-frequency power to high-frequency power.

**Figure 2:**
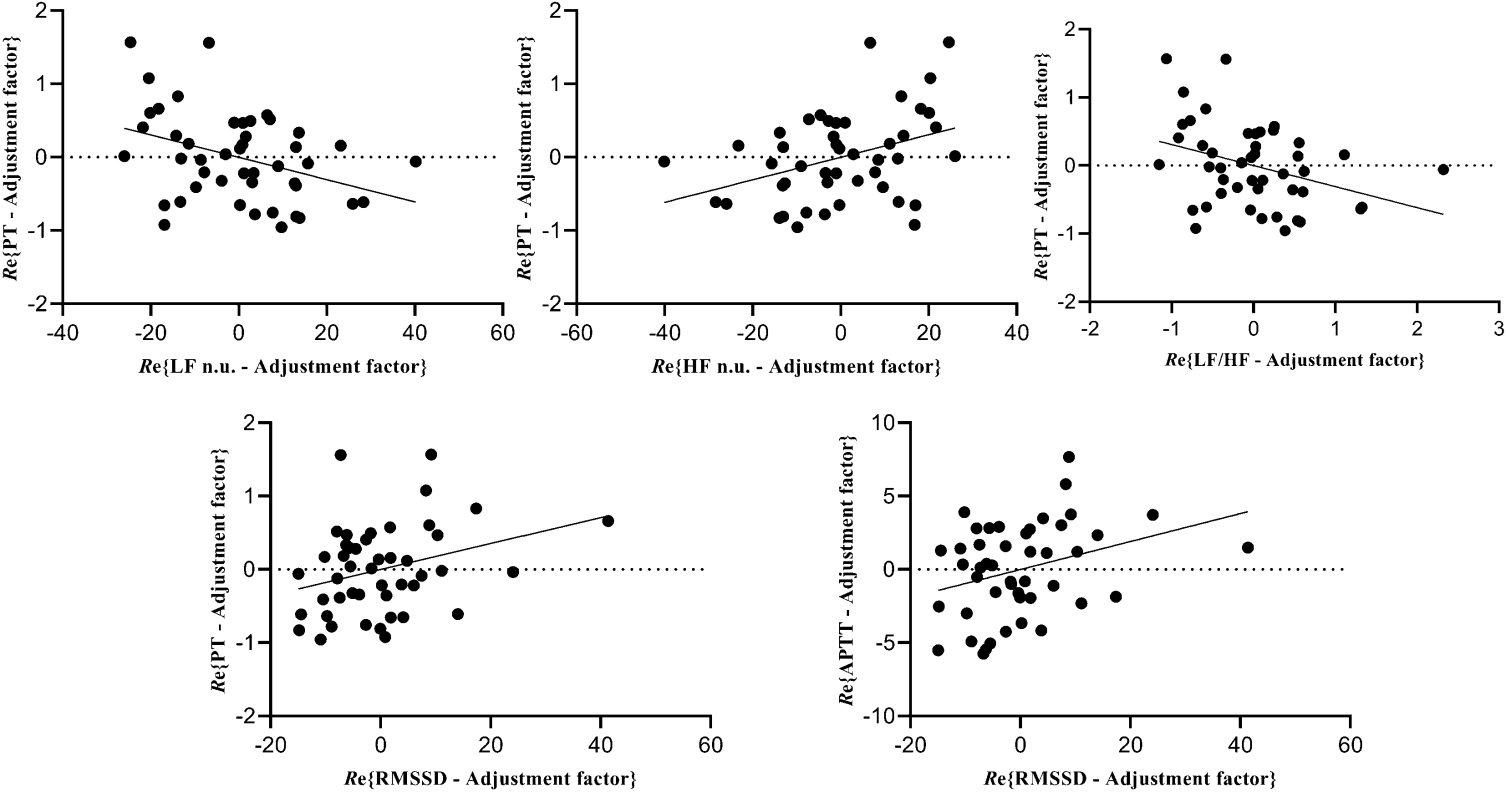
Partial correlation plots of the relationship between RCIs (PT and APTT) and HRV parameters (RMSSD, LF n.u., HF n.u., and LF/HF). Re{y ∼ x} indicates the residuals of regressing y against x.

The results of the bivariate correlation analysis are shown in Table 2. PT was positively correlated with RMSSD, HF and HF n.u. and was negatively correlated with LF n.u. and LF/HF (all *P* < 0.05). Besides, APTT was also positively correlated with RMSSD (*P* < 0.05). After adjusting for age and BMI, RMSSD, LF n.u., HF n.u., LF/HF were still significantly associated with PT. Specifically, the value of PT increased by 0.192 ± 0.091 or 0.231 ± 0.088 s, respectively for each 1 standard deviation (SD) increase in RMSSD or HF n.u.; it increased by 0.230 ± 0.088 or 0.215 ± 0.088 s, respectively for each 1 − SD decrease in LF n.u. or ln (LF/HF) [all false discovery rate (FDR)-corrected *P* < 0.05; Table 3]. RMSSD was significantly associated with APTT, and specifically, for each increase of 1-SD in RMSSD, the value of APTT increased by 1.032 ± 0.470 s (FDR-corrected *P* < 0.05; Table 3). Figure 2 shows the partial correlation plot between RCIs and HRV parameters after accounting for age and BMI.

**Table 2:** Bivariate correlation between HRV parameters and RCIs.

	PT	TT	APTT
SDNN	(0.203, 0.176)	(**− **0.137, 0.362)	(0.200, 0.183)
RMSSD	**(0.331, 0.025)**	(**− **0.146, 0.332)	**(0.308, 0.038)**
LF	(0.111, 0.464)	(0.028, 0.856)	(0.167, 0.267)
HF	**(0.292, 0.049)**	(**− **0.076, 0.617)	(0.259, 0.082)
LF n.u.	**(− 0.395, 0.007)**	(0.197, 0.190)	(**− **0.199, 0.185)
HF n.u.	**(0.397, 0.006)**	(**− **0.192, 0.202)	(0.202, 0.178)
LF/HF	**(− 0.368, 0.012)**	(0.179, 0.234)	(**− **0.221, 0.140)

*PT* prothrombin time, *TT* thrombin time, *APTT* activated partial thromboplastin time, *SDNN* standard deviation of normal-to-normal intervals, *RMSSD* the root mean square of successive heartbeat interval differences, *LF* low-frequency power, *HF* high-frequency power; *LF n.u.* low-frequency power in normalized units, *HF n.u.* high-frequency power in normalized units, *LF/HF* the ratio of low-frequency power to high-frequency power.

Values are expressed as (*r*, *P*). Bold text indicates statistical significance at *P* < 0.05.

**Table 3:** Results of linear regression models (adjusted for age and BMI).

Outcome	Predictor	Coefficient (estimate ± SE)*	*P*	Rank	Q	*P* < Q?
PT	HF n.u.	0.231 ± 0.088	0.012	1	0.008	TRUE
PT	LF n.u.	− 0.230 ± 0.088	0.012	2	0.017	TRUE
PT	LF/HF	− 0.215 ± 0.088	0.019	3	0.025	TRUE
APTT	RMSSD	1.032 ± 0.470	0.034	4	0.033	TRUE
PT	RMSSD	0.192 ± 0.091	0.040	5	0.042	TRUE
PT	HF	0.169 ± 0.094	0.080	6	0.050	FALSE

*SE* standard error, *PT* prothrombin time, *APTT* activated partial thromboplastin time, *RMSSD* the root mean square of successive heartbeat interval differences, *HF* high-frequency power, *LF n.u.* low-frequency power in normalized units, *HF n.u.* high-frequency power in normalized units, *LF/HF* the ratio of low-frequency power to high-frequency power.

*Effects for a 1 − SD increase in the predictor adjusted for covariates. Q: the Benjamini–Hochberg critical value [= (rank/number of tests) × FDR]. TRUE for *P* < Q indicates statistical significance after correcting for the FDR.

## Discussion

Correlation between short-term HRV parameters and RCIs was observed in this study involving 46 BC patients. Specifically, PT was positively correlated with RMSSD and HF n.u. and was negatively correlated with LF n.u. and LF/HF. Moreover, and APTT was significantly positively correlated with RMSSD. However, we did not find an association between HRV and TT in patients with BC.

RMSSD and HF n.u. are considered to reflect parasympathetic nerve (vagus nerve) modulation^22^. The interpretation of LF, LF n.u. and LF/HF is controversial. LF is earlier considered to be related to SNS activity and is more recently believed to mainly reflect baroreceptor reflex activity^23,24^. As a result, the opinion that LF n.u. or LF/HF represents sympathetic-vagal balance has also been challenged. The interaction between sympathetic and vagal modulation is likely complex and nonlinear in nature that also challenges the interpretation and assessment of their balance^25^. APTT and PT are indicators reflecting endogenous and exogenous coagulation pathways, respectively, which can be used to determine the content and activity of coagulation factors in the blood^26^. Therefore, our results directly support the relationship between ANS and coagulation in BC patients.

Autonomic nervous function is closely related to the tumorigenesis and development of cancer^27–29^. There is growing evidence that vagus neuromodulation in tumorigenesis is partially mediated by its anti-inflammatory effect through the cholinergic anti-inflammatory pathway^30^. Specifically, the acetylcholine produced by the vagus nerve can bind to the α-7 nicotinic acetylcholine receptor expressed on tissue macrophages and inhibit the release of inflammatory cytokines through signal transduction of receptors, such as interleukin-1 (IL-1), interleukin-6 (IL-6) and tumour necrosis factor-α (TNF-α), blocking the interaction between tumour cells and the surrounding inflammatory microenvironment^31,32^. The role of vagal activity in regulating tumour progression has also been shown in several human and animal studies. For example, in patients with various cancers, including colon cancer (CC), prostate cancer (PC) and GC, high vagus nerve activity, as indicated by HRV, significantly predicts lower levels of tumour inflammatory markers^33–35^. In vagotomised animals, enhanced tumour metastasis and worse prognosis was also observed^36^.

Patients with malignant tumours usually demonstrate aberrant activation of the coagulation system^37^. Many studies have shown that coagulation disorders caused by tumours not only can promote tumour progression, invasion, angiogenesis and metastasis, but also are closely related to tumour stage and prognosis^1,38^. In fact, coagulation abnormalities in cancer patients, including prolongation or shortening of APTT or PT, thrombocytosis, hyperfibrinogenaemia and elevated D-dimer levels, have been confirmed in different cancers, such as GC, lung cancer, BC, or CC^39–42^. APTT and PT are the two most commonly used RCIs in laboratory examinations, and their prognostic value for cancer has been reported. For example, Qi et al*.* found that the level of PT in patients with non-small-cell lung cancer was significantly higher than that in healthy people, and the survival rate of patients with prolonged PT was significantly lower^43^. Similarly, Wang et al*.* reported that elevated PT level was an effective predictor of survival for cholangiocarcinoma patients which was independent of age, tumour differentiation, and TNM stage^44^. In BC patients, the shortening of PT and APTT directly promoted thrombosis, which facilitated infiltration and metastasis of tumour cells and was significantly related to poorer prognosis of patients^4^.

The activation of coagulation cascades is mainly caused by tissue factor (TF) expressed and released by tumour cells^45^. This transmembrane glycoprotein can form a complex (TF/FVIIa) with coagulation factor VIIa and trigger coagulation by activating coagulation factors IX and X. TF is the main initiator of the coagulation pathway^46^. Studies have shown that in addition to activating blood coagulation, TF also plays an important role in tumour progression and is an important mediator between coagulation, inflammation, thrombosis, tumour growth and metastasis^47,48^. Inflammatory cytokines, including TNF-α and IL-1b, can induce tumour-associated macrophages and endothelial cells to express TF. In addition, these inflammatory factors downregulate important physiological anticoagulant pathways through a variety of mechanisms, which together with the upregulation of TF lead to the procoagulant effect^49,50^. The vagus nerve has a regulatory effect on inflammation, while the anti-inflammatory effect inhibits the expression of TF on monocytes^51^. Therefore, the vagus nerve may inhibit the expression of TF by regulating inflammation to achieve the effect of inhibiting coagulation. Future studies are warranted to further elucidate this mechanism.

In conclusion, previous studies showed that vagus nerve modulation was associated with cancer prognosis. Erin et al. showed that anti-inflammatory drugs that activate the vagus nerve reduced tumor metastasis in tumor-bearing mice^52^. In addition, several studies have shown that higher vagal modulation measured by HRV is associated with longer survival and lower level of C-reactive protein^53,54^. Our study extends the results of previous studies, associating vagal modulation (i.e., RMSSD, HF n.u.) with RCIs, and reveals the inhibitory effect of vagus nerve on blood coagulation in BC patients. It is thus of great significance to understand the beneficial role of the vagal activity in cancer. Further studies are warranted to examine whether an intervention on vagal activity can prevent coagulopathy, slow down the progression of cancer, and improve the prognosis of patients. Besides, the physiological mechanism connecting the ANS with coagulation function is unclear. Surgery, radiotherapy, chemotherapy or other treatments may also change the coagulation function of BC patients^55,56^. How the associations between RCIs and HRV change during and after the treatment in BC patients also required further elucidation.
